# Exploring self-detection of the endogenous LH surge using a urine test as a tool to predict a suboptimal response to gonadotropin-releasing hormone agonist trigger during *in vitro* fertilization cycles

**DOI:** 10.5935/1518-0557.20220047

**Published:** 2023

**Authors:** Maria Martinez, Ricardo Delgado, Estibaliz Quevedo, Jaime Guerrero, Helena Moragues, José A Ortiz, Andrea Bernabeu, Juan Carlos Castillo, Rafel Bernabeu

**Affiliations:** 1 Departament of Reproductive Medicine. Instituto Bernabeu. Alicante, Spain; 2 Departament of Reproductive Medicine. ACCUNA. Alicante, Spain; 3 Master in Reproductive Medicine. Universidad de Alicante, Spain; 4 IBBIOTECH. Alicante, Spain

**Keywords:** GnRH agonist trigger, urine LH test, oocyte donation, trigger failure, empty follicle syndrome

## Abstract

**Objective:**

Is self-detection of the endogenous LH surge using a urine testing a reliable method to confirm a successful gonadotropin-releasing hormone agonist (GnRHa) trigger in IVF cycles?

**Methods:**

Prospective observational study including a total of 103 oocyte donation cycles between November 2019 and January 2020. Urine LH testing (Akralab SL, Spain, cut-of value 30 mIU/mL) was performed at home in samples from the first micturition in the morning after the GnRHa trigger and a picture of the result was sent to the nurse coordinator; this information was concealed and only disclosed after oocyte aspiration.

**Results:**

From the total group, two cycles were excluded. A total of 101 oocyte donors performed the LH urine testing, all proceeded to oocyte aspiration and were included in final analysis. A total of 85 (84.2%) had a positive LH test and an uneventful oocyte retrieval with good retrieval rates (false positive rate: 0%). A total of 16 had a negative LH test (15.8%) and had a good oocyte retrieval rates (false negative rate: 15.8%). There were no cases of empty follicle syndrome.

**Conclusions:**

Due to a high false negative rate, self-testing of endogenous LH release using a LH urine test when performed approximately 12-hours after triggering does not seem to be a reliable method to predict a suboptimal response to gonadotropin-releasing hormone.

## INTRODUCTION

In assisted reproductive technology, the purpose of ovarian stimulation is to obtain multiple oocytes at follicular aspiration. The process of ovarian stimulation is completed with the induction of the final oocyte maturation using human chorionic gonadotropin (hCG) or a bolus of a gonadotropin-releasing hormone agonist (GnRHa). The mechanisms by which a GnRHa triggers the final process of oocyte maturation seems to be determined by the resulting endogenous LH/FSH rise and activity (Chen *et al*., 2012).

A single bolus of GnRHa for final oocyte maturation has been proposed as an effective strategy to prevent OHSS and as a ‘gold standard’ trigger agent for the oocyte donor (Castillo *et al*., 2020). Nonetheless, despite data on its efficacy and safety, it has been recognized that in a small subset of patients, the GnRHa may not elicit a sufficient LH surge to reinitiate meiosis, thus leading to oocyte retrieval failure. This failure can range from empty follicle syndrome (Castillo *et al*., 2012) to the aspiration of lower than expected number of oocytes from the number of adequate size follicles on the triggering day (Meyer *et al*., 2015). In oocyte donation cycles this outcome is notably worrying for the potential implications to both the oocyte donor (exposure to a surgical intervention with absent or limited chances of success) and recipient (cycle cancellation).

A suboptimal response to gonadotropin-releasing hormone agonist trigger during *in-vitro* fertilization cycles is a condition of uncertain etiology. However, human error in the administration of the medication or patient compliance may play a potential role, particularly in oocyte donors. Some studies advocate blood LH measurements at different points during the ovarian stimulation process (Meyer *et al*., 2015; Popovic-Todorovic *et al*., 2019) as means to predict a suboptimal response. Nonetheless, from a practical point of view, this strategy adds inconvenience for the donor.

Early studies described that the GnRHa induced LH surge consists of two phases: a short ascending limb (LH peak ± 4h) and a long descending limb (±20 h), in total ~24–36h (Itskovitz *et al*., 1991). Therefore, the measurement of serum LH post-trigger has been suggested to prevent suboptimal LH rise leading to failure in oocyte retrieval. Considering the post-GnRHa LH pharmacodynamics, most studies test LH levels ±12 hours after triggering. As examples, Kummer *et al*. (2013) detected low LH values as <15 mIU/ml 10 h after trigger in seven cases of EFS among 508 patients triggered with GnRHa. Chen *et al*. (2012) showed that serum LH level at 12-h post-trigger with GnRHa <15.0 IU/l is associated with a dramatically lower oocyte yield. After a GnRHa trigger, the subsequent LH peak lasting for ~24-36 h can be measured not only in serum but also opens the possibility for using an LH surge test in urine as a simple method to confirm whether LH rise has been elicited by the GnRHa. The aim of our study was to explore self-testing of endogenous LH release using a LH urine test 12-hours after triggering as a method to predict a suboptimal response to gonadotropin-releasing hormone agonist trigger in oocyte donation cycles defined as the retrieval of zero oocytes (empty follicle syndrome) after an uneventful ovarian puncture for egg-collection.

## MATERIAL AND METHODS

This is a prospective observational study including a total of 103 oocyte donation cycles performed at Instituto Bernabeu - Alicante in Spain between November 2019 and January 2020. The study was approved by the institutional review board of Instituto Bernabeu Alicante (MR-23/2019).

A total of 103 patients from the oocyte donation program were included in the study. The oocyte donors were 18 – 33 years old with a BMI between 18-29 Kg/m^2^. The blockage of a premature LH peak was achieved using 200 mg of natural micronized progesterone (Progefk®, Efk) once daily per os commencing together with the ovarian stimulation (Castillo *et al*., 2018) in the vast majority of cycles and only a few received daily 0.25 mg GnRH antagonist subcutaneous injection of ganirelix (Orgalutran®, Merck Sharp & Dohme) starting with a leading follicle of ≥ 14 mm. Donors initiating in a “random-start” protocol (irrespective of the day of the menstrual cycle) performed a basal scan control and commenced gonadotrophins concomitantly with progesterone. Only donors commencing the ovarian stimulation on day 1-3 of the cycle (deemed “conventional” initiation of ovarian stimulation) proceed with a basal scan evaluation but did not receive progesterone for LH peak suppression. A second scan control was scheduled 5/6 day later and every 2-3 days thereafter until achieving the criteria to induce the final follicular maturation. A bolus of triptorelin 0.4mg (Decapeptyl® 0,1 mg, Ipsen Pharma, Spain) was used to induce the final oocyte maturation when >2 follicles >17 mm were visualized. Oo-cyte aspiration was performed 36-hours after the administration of the GnRHa trigger.

### Protocol for GnRHa administration

Triptorelin was prepared and mixed (four vials of powder + 2 ampules of diluent) by a trained nurse and then provided to the oocyte donor in a single mixed syringe; thus, the solution was ready for self-subcutaneous injection at the appropriate time. Careful instructions were provided to the donor to keep the solution in a cool environment until administration. A notification was sent to the on-call nurse coordinator as soon as the GnRHa triggering was carried out. The goal of all these steps was to minimize potential human errors or loss of the solution during reconstitution of the medication. It is worth noting that this is the standard protocol for GnRHa administration in oocyte donors in daily practice in our centre.

### Protocol for LH urine testing

All donors were instructed by a trained nurse how to perform the urine LH testing (Akralab SL, Spain, sensibility 30 mIU/mL) at their last visit to our clinic. The LH urine test is intended for use in the detection of human LH through visual interpretation of colour development on the internal strip. During testing, the specimen reacts with further anti-hLH antibodies conjugated to coloured particles and precoated on the conjugate pad of the internal strip (immunochromatography). If there is sufficient hLH in the specimen, a coloured line appears in the test line region (LH) of the membrane. The manufacturer states that the presence of this line with the same or stronger colour intensity than that of the control line (C) indicates a positive result. As the cut-of value below which it has been shown that there is risk of retrieving less oocytes than expected is <15 mIU/mL and our urine test has a sensibility of 30 mIU/mL, we considered only a negative value if there is no visible line in the urine test. Self-testing was scheduled with micturition the morning after triggering (12-hours post GnRHa administration) and a digital picture of the result was sent via WhatsApp (WhatsApp Ireland Limited, Dublin, Ireland) application for smartphone as soon as the test was performed to one specific and trained nurse coordinator who oversaw: receiving, visually interpreting, tracking and concealing the results. The nurse coordinator was blind to the outcome of the egg retrieval. Conversely, the physician in charge of performing egg retrieval and embryologist staf were blind to the results of the test. Test results were disclosed only after concluding the study. Thus, no additional tests for confirmation of LH levels or ‘rescue’ protocols were scheduled in the eventuality of a negative LH urine test.

### Statistical analysis

The normality in the distribution was evaluated through Shapiro-Wilk’s test. For the statistical analysis, numerical variables normally distributed were presented as mean, standard deviation and range and numerical variables not normally distributed were presented as median, IQR (interquartile range) and range. A *p*<0.05 value was considered as statistically significant after performing a t-student test (parametric) or Mann-Whitney U test (non-parametric). Categorical variables were presented as number of cases and percentage. The statistical analysis was performed with the software SPSS 20.0 (SPSS, Chicago, IL, USA).

## RESULTS

A total of 103 oocyte donors were included in the study period, two were excluded due to concomitant participation in another trial potentially influencing the results. A total of 101 oocyte donors performed the LH urine test and proceed to a transvaginal oocyte aspiration, these 101 oocyte donors were included in the final data analysis. A total of 85 oocytes donors had a positive urine LH test (84.2%) and an uneventful oocyte retrieval with good retrieval rates (false positive rate 0%). A total of 16 oocyte donors had a negative LH test (15.8%) and had a good oocyte retrieval rates (false negative rate: 15.8%) (Table 1). The results were similar in terms of pre-aspiration parameters (number of follicles ≥ 14 mm the triggering day) or post-aspiration parameters (total number of collected oocytes and number of metaphase II oocytes) between oocyte donors with a positive versus negative urine LH test (Table 2) and their basal characteristics were also similar except for a trend of BMI in the lower range in LH negative patients (Table 3). There were no cases of empty follicle syndrome.

**Table 1:** Basal characteristics oocyte donors.

General demographics	Total	LH positive	LH negative	p value
Age (years)	*25 (8)	*25 (8)	*25 (5)	0.918^a^
BMI (kg/m^2^)	*22.57 (4.17)	*22.59 (4.37)	*21.08 (3.55)	0.060^a^
AFC	*15 (6)	*15 (7)	*16 (7)	0.223^a^
Days of stimulation	*10 (3)	*10 (3)	*9 (3)	0.226^a^
Total gonadotropin	**2243.76 (720.82)	**2304.21 (709.94)	**1913.33 (713.01)	0.056^b^
Number of previous cycles	*1 (4)	*2 (4)	*1 (6)	0.741^a^

a Man-Whitney U Test

b Student T test

* Median (IQR)

** Mean(SD)

**Table 2:** Data for LH urine test results.

	Frequency (%)	Estimates (95% CI)
Negative	Frequency	Estimates False
Negative	16 (15.8)	15.8% (9.993 - 24.194)
Positive	85 (84.2)	Sensibility: 84.15% (75.8 - 90)
Total	101 (100)	

**Table 3:** Pre and post follicular aspiration parameters according to LH Urine Test results.

Parameters	LH Urine Test results						
	Negative (n=16)			Positive (n=85)			p-value*
	Median	IQR	Range	Median	IQR	Range	
Follicles >15 mm at trigger	12	6	(8-19)	10	6	(3-22)	0.154
Aspirated oocytes	14	10	(8-36)	13	10	(3-38)	0.506
Metaphase II oocytes number	12	7	(7-35)	11	9	(2-37)	0.520

* Mann-Whitney U test.

## DISCUSSION

In oocyte donation cycles, an inadequate response from the hypophysis to a GnRH agonist trigger is an infrequent but challenging situation with potentially signif-cant consequences. The presentation of this event varies in the medical literature but it is estimated to happen in approximately 3% of oocyte donation cycles (Castillo *et al*., 2012); although the exact etiology is still elusive, involuntary errors in the administration of the medication or patient compliance (particularly in oocyte donors) may play a role. Ultimately, no definitive method for a practical, non-demanding and effective prevention exists.

Some studies advocate LH testing in blood at the start of the stimulation, on the day of trigger or the morning after the trigger (Meyer *et al*., 2015; Popovic-Todorovic *et al*., 2019) as means of predicting a suboptimal response to GnRHa trigger. Nonetheless, from a practical point of view this is inconvenient for the patient/donor as it adds extra visits to the clinic and /or extra blood extractions. Recently, LH surge self-testing in urine has been described as a simple and cheap method to confirm that LH rise was induced by a GnRHa trigger in the oocyte donor population (Cozzolino *et al*., 2020). However, our results challenge this initiative as a reliable method for confirming an adequate LH rise post GnRHa trigger.

A strong point from our trial is that the results from LH urine were disclosed only after oocyte aspiration, allowing for an accurate evaluation about the performance of test in a real-life scenario and avoiding an additional source of potential bias. In the current study, a vast proportion of LH urine test were positive, and no false positive cases were found. Nevertheless, 15.8% of LH urine tests were negative using a standard and commercially available test, and still in all these oocyte donors good retrieval rates after puncture were achieved.

LH urinary tests are produced for detection of spontaneous LH surge. Peak LH following agonist trigger reaches the same amplitude of the spontaneous LH surge, however, it lasts for a shorter time. Therefore, the “area under the curve” is very short, setting the stage for high false negative rate, as encountered in our study. Importantly, this implies that if we had relied on the results from the test, a considerable proportion of oocyte donors would have undergone additional and unnecessary tests/visits to the centre for further confirmation as a consequence of a false negative test. Moreover, if a ´rescue´ protocol using hCG as re-trigger agent would have been established based solely on the result from the urinary LH test, a sig-nificant proportion of oocyte donors would have been erroneously submitted to a potentially dangerous strategy and yet the outcome in terms of the oocyte collection would have been compromised.

In the previous study (Cozzolino *et al*., 2020) a false negative of only 0.85% (3/371) was found when using a similar urine LH test for confirming LH rise the morning after triggering. Several factors may account for this evident discrepancy. First, the minimum detection level for the test employed by Cozzolino *et al*. (2020), was 25 mIU/mL as opposed to a level of 30mIU/mL for the test employed in our study. Even though an exact cut-of level below which a failure in oocyte collection after GnRHa trigger is expected is still a matter of research, some studies advocate a cut-of of 15 mIU/mL in blood samples (Kummer *et al*., 2013). This implies that even if not detected by the urine test, serum LH values may still be good enough allowing for an adequate oocyte collection after triggering and this proportion of false negative cases tends to be higher in correlation with the minimum threshold for detection in the LH urine test, as suggested from our results. With the usual detection limit for home urine ovulation prediction kits (20–40 mIU/mL), false-negative results may occur in case of diluted urine and/or LH surges of short duration or with low peak values, as suggested by others (Zreik *et al*., 1999; Mitwally *et al*., 2004). It is has to be reminded that the LH surge produced after triggering with GnRH agonist is similar to the one in natural cycles (in which ovulation prediction kits were studied) when comparing peak values but it is shorter in duration (Castillo *et al*., 2020) being this a possible cause for a false negative result. Consequently, an initial negative urine LH test must be followed by an LH blood test for confirmation as we have seen in a recent publication in oocyte donors (Massin, 2017). All in all, these findings suggest that, ideally, tests with lower minimum detection limits must be employed if self-testing in urine should be used for ascertaining that an endogenous LH surge was efficiently induced by a GnRHa trigger in order to prevent oocyte retrieval failures and even these tests with lower minimum detection limits must be confirmed (LH testing in blood) before being used for this purpose in clinical practice. A final point to take into consideration on the subject is that our false positive rate was determined by having no empty follicle syndrome in the studied population which could be explained by the low incidence of this event (0.59%-3.5%) in IVF cycles (Castillo *et al*., 2020).

Involuntary errors in the administration of the medication (particularly in oocyte donors) are always a point of concern. In order to decrease a potential human error and at the same time to facilitate the management of the medication, in our centre the GnRH agonist is mixed and pre-filled by a nurse and only then given to the oocyte donor in an individual syringe for self-administration at the appropriate time. This methodology introduces a second factor of diference with the study by Cozzolino *et al*. (2020), for instance, we can hypothesize that a decrease in the biopotency of the agonist could be present if there is a delay from mixing triptorelin solution up until the administration of the medication, still potent enough in order to elicit a proper oocyte maturation (Zelinski-Wooten *et al*., 1991), but with a rapid decline thereafter, favoring lower endogenous LH concentration when tested 12-hours apart in urine. Even though plausible, our data does not show correlation between longer times between mixing and administration (41.5%, 42/101 oocyte donors received the mixed solution the day before the injection) and lower endogenous LH activity if measured by the number of collected oocytes in the false negative donors group (data not shown); however the sub-group numbers are small in order to make definitive conclusions on this subject.

As a final note for future studies and based on the pharmacodynamics of the GnRH agonist as trigger agent, it would be interesting to explore the efficacy of LH urine tests measured 4-hours after GnRHa administration (peak LH release after GnRHa triggering) to predict a suboptimal response to a GnRHa trigger.

In conclusion, our results suggest that a urine LH self-testing performed at home by the patient, possess an optimal positive correlation with a successful oocyte collection specially in case of a positive LH urine test. However, the false negative rate of LH urine sticks -requiring additional serum LH confirmation-, limits its applicability in general clinical practice, thus, caution is warranted. Since failure to respond to a gonadotropin-releasing hormone agonist trigger in terms of an adequate LH rise is a relatively rare phenomenon, future larger sample size studies employing LH urine test (preferably) digital readout home urine LH kit with lower minimum threshold detection values are required to establish the real validity of this strategy in in-vitro fertilization cycles.
